# Epitope-Specific Antibody Levels in Tuberculosis: Biomarkers of Protection, Disease, and Response to Treatment

**DOI:** 10.3389/fimmu.2014.00243

**Published:** 2014-06-02

**Authors:** Graham H. Bothamley

**Affiliations:** ^1^Department of Respiratory Medicine, Homerton University Hospital, London, UK

**Keywords:** tuberculosis, epitopes, B-lymphocyte, biomarkers, antibodies, monoclonal, antibody specificity

## Abstract

Monoclonal antibodies restricted to *Mycobacterium tuberculosis* can measure epitope-specific antibody levels in a competition assay. Immunodominant epitopes were defined from clinical samples and related to the clinical spectrum of disease. Antibody to the immunodominant epitopes was associated with HLA-DR15. Occupational exposure showed a different response and was consistent with recognition of dormancy-related proteins and protection despite exposure to tuberculosis (TB). Studies in leprosy revealed the importance of immune deviation and the relationships between T and B cell epitopes. During treatment, antibody levels increased, epitope spreading occurred, but the affinity constants remained the same after further antigen exposure, suggesting constraints on the process of epitope selection. Epitope-specific antibody levels have a potential role as biomarkers for new vaccines which might prevent the progression of latent to active TB and as tools to measure treatment effects on subpopulations of tubercle bacilli.

## Introduction

There are many unanswered questions in tuberculosis (TB) for which an understanding of both clinical aspects and the adaptive immune response is critical. Most research has concentrated on the processes of infection and the initial, innate immune response to *Mycobacterium tuberculosis* (Mtb). It has long been clear that BCG vaccination is excellent at preventing primary forms of TB and that the immunodeficiencies caused by HIV infection or by increasing age give rise to these same forms of TB. On the other hand, BCG does not prevent post-primary disease, particularly sputum smear-positive pulmonary tuberculosis (S + PTB). Many animal models which attempt to elucidate the nature of reactivation of latent infection merely recapitulate the same pattern of immunodeficiency found in primary disease.

Post-primary TB is characterized by an immune response to both cross-reactive antigens, as in the tuberculin response, and species-restricted antigens, such as those found in the RD1 sequence, namely esat-6 and cfp-10. Destructive caseation is an essential feature of post-primary disease and much has been made of the difference between apoptotic and necrotic cell death as the pathogenetic mechanism (1). HIV infection has shown that CD4+ T cells are essential in this process, as lung cavities become rarer as the CD4 count falls (2). Sette et al. observed that antigen concentration was important in predicting T helper responses and that antibody responses reflected both the CD8 T cell response to early antigens and the CD4+ T cell response to late and structural antigens (2).

This paper will describe the data available on antibody responses to species-restricted B cell epitopes according to clinical parameters. It will explore whether these immunological markers can discriminate among the clinical states of TB infection and disease. Underlying this discussion remain the problems of why some B cell epitopes are immunodominant, how antibody diversity becomes fixed, whether conformational epitopes are more important than linear epitopes, and the relationship between T and B cell epitopes.

## The Measurement of Epitope-Specific Antibody

A soluble extract from irradiated Mtb, prepared by crushing with glass beads or ultrasonic degradation, gave a better range of antigens than tuberculin (4). Mouse monoclonal antibodies (Mabs) were created by inoculation with either Mtb or its soluble extract and tested for specificity to Mtb (4). Competition with human sera was tested using labeled Mabs (5), or by exploiting the difference between mouse and human heavy chains in an ELISA (6).

## Lipoprotein Antigens of *Mycobacterium tuberculosis*

The importance of lipoproteins to the immune response has been demonstrated by deleting the prolipoprotein signal peptidase (IspA) (7). S + PTB is characterized by higher levels of antibody to mycobacterial antigens than are other forms of TB with smaller bacterial loads. More than 80% of patients with this form of disease recognize epitopes of the 38-kDa lipoprotein antigen (Rv0934, Antigen 5, Antigen 78, PstS1, PhoS) and epitope-specific antibody correlates well with antibody levels to the purified antigen (8–12). The extent of pulmonary disease has shown a positive association with IgG antibody to the 38-kDa antigen, levels of which were also higher in the few who died from TB (13). In S + PTB, there is a clear association with HLA-DR15, which is also associated with higher anti-38-kDa antibody levels (noting that the control population in this study was from a high incidence area and the majority were nurses on a TB ward who were regularly exposed to Mtb but who did not develop disease) (14). One suggestion is that TB-associated HLA phenotypes have more focused epitope recognition of TB antigens than those associated with healthy controls (15). Another possibility is that this antigen preferentially provokes a pro-inflammatory (Th17) immune response (16). In this context, it should be noted that the same HLA association has been found with S + PTB in other studies in different populations (17, 18), but more importantly that this association was not found in those with smear-negative pulmonary disease (19).

Epitope-specific (TB23 Mab) and antibody levels to the purified 19-kDa antigen (Rv3763, LpqH) contributed most to improving the serological sensitivity after the 38-kDa antigen in a study of six Mabs and human sera (12). Antibody levels to the purified antigen were more important than its TB23 epitope in the diagnosis of extra-pulmonary TB (20). Analysis of human T cell epitopes showed that p61–80 peptide was important, especially in patients with lymph node TB (21, 22).

HIV–TB co-infection has demonstrated three facts about the role of CD4+ T cells in TB (2). Firstly, they prevent the reactivation of latent TB. Secondly, they prevent disseminated disease and their loss is associated with primary TB. Thirdly, they have a pathogenetic role in cavitation, as cavities and significant pulmonary infiltration become rarer as the CD4 count falls. The majority of proteins secreted by actively dividing bacteria and recognized by polyclonal sera are fibronectin-binding proteins, such as the antigen 85 complex, but this group includes the 38-kDa antigen (23). Thus, these antigens are likely good serodiagnostic agents for infectious TB but poor candidates for vaccines as their recognition occurs at the same time as cavitary lung disease.

Children with TB and those with extra-pulmonary (EP) TB did not have antibody to the 38-kDa antigen (5). Patients with TB meningitis, of whom 90% have no pulmonary focus, also did not have anti-38-kDa antibody (24), nor did other patients with primary TB (20). Studies of contacts of TB did indeed show that antibody level to one of the 38-kDa epitopes (defined by TB72) could be found at low but measurable levels in those with a statistically high probability of being infected (25). Perhaps early recognition of the 38-kDa antigen indicates a subject more likely to develop infectious S + PTB.

## Dormancy-Related Antigens

One of the surprising findings in measuring epitope-specific antibody levels was the presence of antibody to the TB68 epitope of the 16-kDa antigen (Rv2031, 14 kDa, hsp16, hspX, Acr) in nurses on a TB ward with frequent exposure to infectious TB (26). The bacterial load was estimated to be small in such healthy individuals and, according to the Th1/Th2 hypothesis, the response in such individuals should have been of cellular rather than humoral immunity. Although this epitope was species-restricted, it was not the immunodominant epitope of the purified antigen. Antibody to the whole antigen was associated with a better prognosis, self-healed disease, and more limited pulmonary disease (13). T cell responses to the 16-kDa p21–40 and p111–130 peptides were also more likely in those with BCG vaccination or EPTB compared to S + PTB (27).

## Epitope-Specific Antibody in Leprosy

A series of Mabs which reacted to lipoarabinomannan (LAM) were able to define two groups, one which reacted equally with LAM derived from both Mtb and *Mycobacterium leprae*, and one series which bound predominantly to *M. leprae* (28). The structural basis for these epitopes was determined by noting the predominance of mannose capping of LAM in Mtb (29) and using knockout mice for *embA, embB*, and *embC* together with competitive binding to synthetic carbohydrates (20, 30, 31). Sera from TB patients showed no binding to the leprosy-specific epitope (unpublished data, using the Mabs ML02 and ML34).

However, for protein antigens, antibody to the Mtb-specific epitopes could be detected in sera from patients with leprosy, although no antibody to the leprosy-specific ML04 epitope (35-kDa antigen) was found in TB patients (32). Two explanations exist for this finding. Firstly, shared T cell epitopes between homologous proteins in the two mycobacterial species might “help” B cells, which had originally been stimulated in response to previous TB infection. Secondly, the antibody epitopes on homologous proteins of *M. leprae* might overlap the binding site of the Mtb-specific Mabs sufficiently to inhibit binding, there being no homolog of the *M. leprae* 35-kDa antigen in Mtb. Bystander stimulation of B cells seems less likely although the probability of exposure to leprosy would have been less than TB (33). Conformational B cell epitopes are flat, oblong ovals with hydrophobic amino acids at the center surrounded by a halo of charged residues (34). Thus, antibody epitope cross-reactivity is unlikely. The effect of trapping of antigen by surface immunoglobulin influences the T cell repertoire (35) and cryptic T cell epitopes may be revealed (36).

## Epitope-Specific Antibody during TB Treatment

Antibody levels are proportional to antigen levels and strong T follicular helper cell responses can often initiate bystander B cell activation hypergammaglobulinemia (3, 33). Patients with TB characteristically have hypergammaglobulinemia. An early finding in the quest for a serodiagnostic test for TB was that antibody levels rose during treatment. This meant that evaluation of tests required pre-treatment sera. In a detailed study of sera from 40 TB patients during treatment (37), antibody to LAM showed a single rise and fall in antibody titer, whereas anti-protein antibody had an early rise within the first 2 weeks of treatment followed by a fall and a second rise during the continuation phase of treatment. This would be consistent with killing of different populations of tubercle bacilli, as suggested from chemotherapy trials by Mitchison (38). The rapidly dividing population is sensitive to isoniazid and standard treatment kills 99% of bacilli in the first 2 weeks. In this study, those with isoniazid-resistant strains of Mtb failed to show the first rise in antibody titers (37).

In an acute inflammation, the immune response tends to be focused on a few immunodominant antigens and a phase of chronic inflammation is associated with epitope spreading (39). Antigen processing by B cells is thought to be important in the process of epitope spreading (36). In cancer immunotherapy, such epitope spreading is associated with a good response to treatment (40). Using vaccinia as a model of complex immune responses, Sette et al. observed that the concentration of antigen was important in predicting T helper responses and that antibody responses reflected both the CD8 T cell response to early antigens and the CD4+ T cell response to late and structural antigens (3). The number of epitopes recognized by TB patients increased with treatment and was especially marked for epitopes other than the immunodominant determinants of the 38-kDa antigen (29, 37).

Following epitope-specific antibody levels during treatment, despite changes in antibody levels, the affinity constant for the antibody or antibodies to an individual epitope did not change (37). In HIV and influenza responses, there appears to be a convergence of epitope recognition, but “deep” sequencing has suggested that this is accompanied by a divergence in the aminoacid sequences forming the antibody binding site (41). This would suggest that affinity should continue to improve and the absence of such a change during TB treatment is therefore surprising and requires further investigation.

Can the changes in epitope-specific antibody levels predict cure or relapse? Preliminary evidence suggests that antibody to the dormancy antigen α-crystallin (TB68 epitope of the 16-kDa antigen) might be helpful in predicting relapse during treatment, but as antibody levels persisted beyond successful treatment, a biomarker of cure still eludes us (37).

## Conclusion

Antigen recognition varies across the TB spectrum. Antigen concentration is likely responsible for the immunodominance of epitopes of secreted proteins in S + PTB. There may be a role for measuring antibody/T cell responses to dormancy antigens and some lipoproteins as predictors of disease and biomarkers of protection and response to treatment.

## Conflict of Interest Statement

The author declares that the research was conducted in the absence of any commercial or financial relationships that could be construed as a potential conflict of interest.
